# Source, distribution, and risk assessment of polycyclic aromatic hydrocarbons in sediment and fish samples from River Owan, Edo State, Nigeria

**DOI:** 10.3389/ftox.2023.1250943

**Published:** 2023-11-27

**Authors:** Akinyinka Akinnusotu, Justina E. Ukpebor, Felix E. Okieimen

**Affiliations:** ^1^ Department of Science Laboratory Technology, Rufus Giwa Polytechnic, Owo, Nigeria; ^2^ Department of Chemistry, University of Benin, Benin City, Nigeria; ^3^ Department of Environmental Toxicology, The Institute of Environmental and Human Health (TIEHH), Texas Tech University, Lubbock, TX, United States

**Keywords:** Eco-toxicology, Risk-Assessment, Owan, PAHs (polycyclic aromatic hydrocarbons), Diagnostic ratio, Sediment

## Abstract

Polycyclic aromatic hydrocarbons (PAHs) are persistent environmental contaminants that present several environmental risks including human health. The 16 priority PAHs including its 1-methylnaphthalene, and 2-methylnaphthalene were determined in sediment and fish samples (*Clarias anguillaris and Oreochromis niloticus*) of River Owan, Edo State, Nigeria using gas chromatography (GC) equipped with flame ionization detector (FID) and other standard laboratory protocols. The isomeric ratio was used for source diagnosis, sediment quality guidelines, and risk models of incremental lifetime cancer were used for risk assessment. 1-methylnaphthalene and 2-methylnaphthalene were most predominant in all sediment samples analysed. The ∑LMW PAHs ranged between 0.093—0.250 μg/kg; ∑HMW PAHs were 0.107—0.579 μg/kg. The sediment samples range for ∑PAHs was 0.280–0.810 μg/kg with concentration order of increase: SE5>SE4>SE3>SE6>SE1>SE2>SE7 for the seven sampling locations. The ∑PAHs for *Oreochromis niloticus* was 0.190 μg/kg, which is higher than the value of *Clarias anguillaris* 0.080 μg/kg, and these values were greatly lesser when compared to the European Commission limit of 12.00 μg/kg. The diagnostic ratio indicates that the sources are more pyrogenic than petrogenic, revealing combustion from grass, wood, and bush burning. Sediment quality assessment showed that the ∑PAHs were lower than the regulatory values of sediment quality guidelines (SQG) assessment suggesting no ecotoxicological effects on the benthic organisms in this area at present. The Incremental Life Cancer Risk results were in the range of 9.15 × 10^−12^—1.46 × 10^−6^ for children, and 7.78 × 10^−12^—1.76 × 10^−6^ for adults considering the three routes of exposure. The incremental life cancer risk assessment showed a negligible risk.

## 1 Introduction

Organic compounds known as polycyclic aromatic hydrocarbons (PAHs) are made up of at least two fused benzene rings arranged in various ways which are produced as a result of carbon-based materials’ incomplete combustion. As a result of their detrimental implications on the health of man, and the environment, which include their carcinogenicity, mutagenicity, teratogenicity, etc., sixteen (16) members of PAHs are documented to be priority contaminants according to the United States Environmental Protection Agency (Barakat et al., 2011; Li et al., 2015; Cui et al., 2016; Adekunle et al., 2018; Kosek and Ruman, 2021; Shariatifar et al., 2021; Ambade et al., 2023; He et al., 2023). The majority of PAHs are generated when organic molecules undergo heat decomposition known as pyrolysis, and then recombination called pyrosynthesis to produce them, e.g., acenaphthylene, anthracene, benz[a]anthracene, acenaphthene, etc. There exist two categories of PAHs which are established by considering the number of benzene rings present in them: low molecular and high molecular weights with the acronym LMW and HMW (Banger et al., 2010; Li et al., 2015; Lawal, 2017; Ambade et al., 2022a; Roy et al., 2022; He et al., 2023).

Due to their high lipophilicity, PAHs are extremely soluble in organic solvents that have fused benzene rings. Each ring structure and isomer of PAHs has a unique UV spectrum due to the compounds’ specific UV absorbance spectrum. The majority of PAHs are fluorescent as well, and when activated, they release certain light wavelengths (Yu et al., 2014; Kuppusamy et al., 2016; Lawal, 2017; Gao et al., 2018). Environmental matrices like soil, mixtures of air, water, biota, sediment, and household products like cosmetics (Wang et al., 2010; Xiao et al., 2012; Adekunle et al., 2018; Wang et al., 2019; Ambade et al., 2021a; Ambade et al., 2021b; Ambade et al., 2023), food produce, and its products (Wang et al., 2017; Sadowska-Rociek et al., 2021; Sharma et al., 2021) are common media with PAHs concentrations that have been established, making them ubiquitous chemicals.

Several activities and processes released PAHs into the surroundings including industrial and domestic activities such as wastewater discharges, oil spillage, asphalt particles, vehicular emissions, and exhausts, washing from oil tanks, leakages from marine vessels, gas flaring, disposal of used petroleum products into the river where adequate environmental management policies are not available, including the burning of fossil fuels, forest fire, etc. (Rogowska et al., 2015; Paloluoğlu et al., 2016; Wang et al., 2017; Sharma et al., 2021; Ambade et al., 2022b). Their routes of exposure are inhalation, ingestion, and dermal absorption; these are the three major ways by which PAHs get into humans. Research has shown that the environment is the primary receiver, and sediment is the major sink and reservoir of PAHs and other environmental pollutants due to the ease of their absorption into organic matter (Srogi, 2007; Feng et al., 2009; Wang et al., 2013; Lang et al., 2015; Olayinka et al., 2019; Ambade et al., 2023). The major ways of PAHs transportation, distribution, and dispersal in the environment include atmospheric transport, erosion, wind, storms, and so on. The buildup of PAHs in sediment and biota causes food chains to become contaminated, which poses a threat to the health of man and animals including biota such as fish (Cui et al., 2023).

According to reports, fish are the most susceptible aquatic organisms for detecting contaminants and pollution in an aquatic environment; hence, they have been used for environmental monitoring of contaminants in rivers (Cui et al., 2016; Olayinka et al., 2019; Pang et al., 2021; Ciucure et al., 2023). Arising from their capacity to bio-accumulate in biota, persist in the environment, having mutagenic and carcinogenic potencies in man, the US EPA has banned some PAHs, hence the interest of all stakeholders especially the researchers for many years (US EPA, 1993; US EPA, 2001; Orecchio and Papuzza, 2009; Lawal, 2017; Gao et al., 2018; Baran et al., 2021; Cui et al., 2023). Several researchers have analyzed, characterized concentrations, and carried out health implications of POPs in which PAHs a member of that group in the past decades across the globe (Lau et al., 2010; Yu et al., 2014; Li et al., 2015; Wang et al., 2017; Gao et al., 2018; Ekere et al., 2019; Usese et al., 2021; Cui et al., 2023).

Considering the dangers PAHs represent to the environment, wildlife, and the health of man, scientific research on them has attracted considerable attention in many developed nations. However, there are significant data gaps regarding PAHs sources and levels in developing nations (Ogunfowokan et al., 2003; Nieuwoudt et al., 2011; Okedeyi et al., 2013; Mirza et al., 2014; Zheng et al., 2020; Sharma et al., 2021; Usese and Egbuta, 2021; Ambade et al., 2023). Several samples including water, soil, sediment, fish, and other aquatic organisms from rivers, oil spillage sites, industrial locations, polluted soil, agricultural land use areas, etc. have been published (Ogunfowokan et al., 2003; Jiao et al., 2013; Asagbra et al., 2015; Edokpayi et al., 2016; Adeniji et al., 2019; Zheng et al., 2020; Sharma et al., 2021; Bajt, 2022; Ambade et al., 2023), there are no reports of studies on PAHs from River Owan and its environments except a preliminary study executed by our group (Akinnusotu et al., 2020). There are rivers in Edo State, Nigeria in which River Owan is one of them with several economic significance. These include various agricultural practices (extensive and intensive farming), irrigation of farms, fishing from the river, lumbering, transportation of goods and services to several camps around the river, marketing of the various farm produce around this area, and so on (Omoregbee and Edeoghon, 2006). Some other human activities around the river include forest and bush burning from farms which are common practices, dumpsites located very close to the river, speed boat engine repair workshops constructed around the river, major markets for the community located close to the river as a meeting point, most drainages in the community are channeled towards the river, transportation of logs of wood, etc. It serves as a primary source of water for the use of the community including drinking, and other domestic uses. Hence, this research provides further data on the concentration of PAHs in River Owan, Edo State, Nigeria by determining its concentrations in fish and sediment samples, identifying its possible sources, and evaluating health risk using risk assessment models.

## 2 Materials and methods

### 2.1 The area of study

**FIGURE 1:**
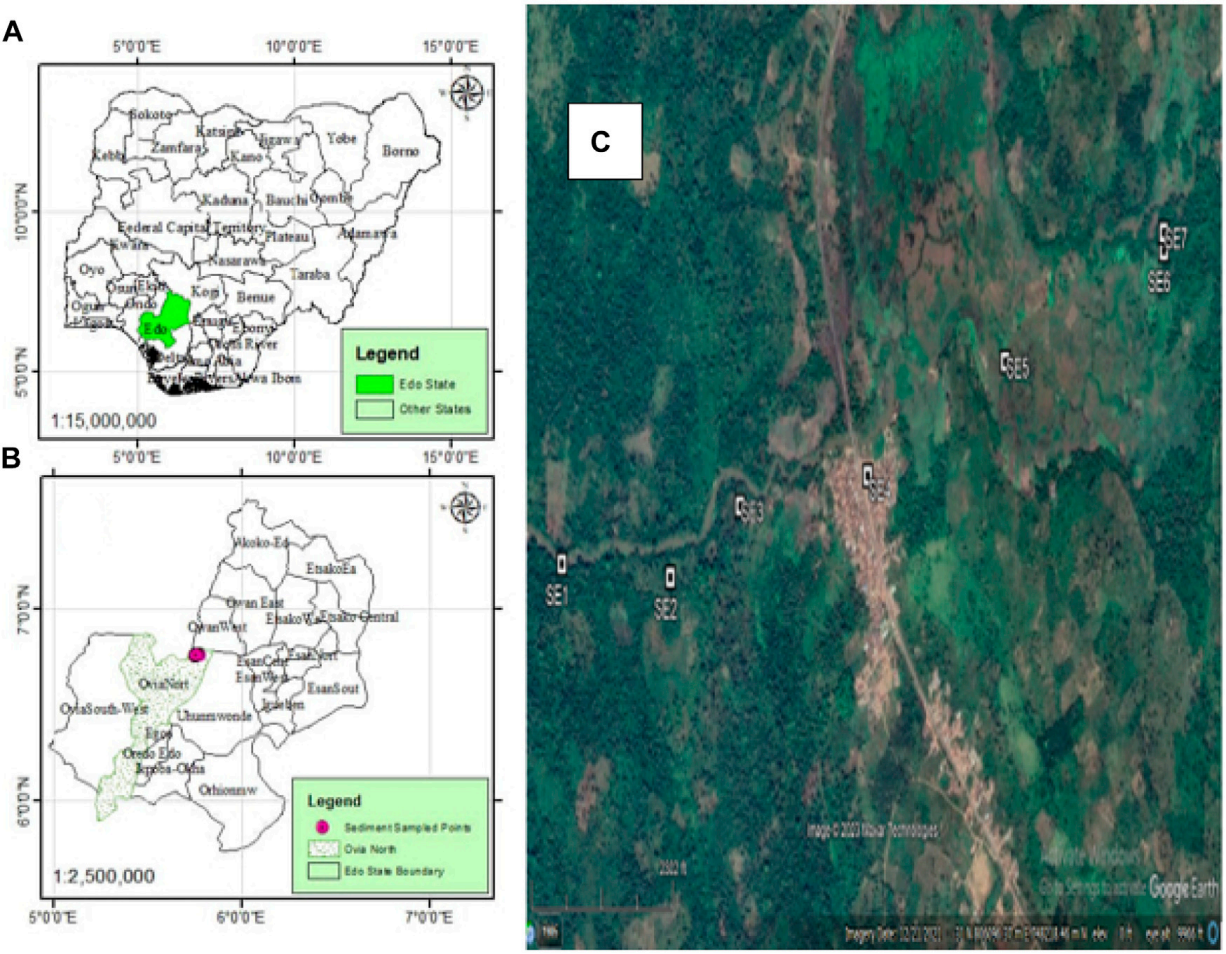
Map of the study area and locations of sampling.

Owan is a community located in Edo State, Nigeria Figures 1A, B with a major River that lies on latitude 06° 45′ 40″ N; and longitude 005° 46′ 07.4″ E (Ogbeide et al., 2015). The two climatic seasons in Nigeria are the dry season and the wet season. The dry season typically lasts from November through March of the year, whereas the wet season is typically between the months of April and October. Some of the camps and communities around River Owan are Agbenikaka, Odei, Ogbigbi, Okpokhumi, Sabongida, etc. The communities are notable for farming activities that involve both cash and food crops. Some of the crops include cocoa, palm tree, plantain, banana, yam, cassava, etc. The river is used for irrigation of close farm plantations, fishing, and domestic use such as washing, cooking, bathing, and drinking. Other activities in the study area include mechanic workshops for the repairs of various automobiles, the burning of wastes, and major markets located along the river banks (Ogbeide et al., 2015; Akinnusotu et al., 2020). Seven sampling locations were used considering human activities, vegetation, and ecological settings that run across the river.

### 2.3 Sampling and sampling locations

#### 2.3.1 Sediment

Samples of sediment were taken across the river from seven different sampling locations using a stainless-steel grab sampler, putting the samples into a 1-L glass jar having a cap (cleaned), and transported to the Chemistry Laboratory at the Rufus Giwa Polytechnic, Owo, Nigeria, in an ice-chest at 4°C. Seven different sampling locations were selected where sediment samples were taken across the river. The sampling locations and samples were assigned codes SE1 to SE7 (Figure 1C). Sampling location coordinates were obtained with the aid of a Garmin GPSMAP 76S GPS.

#### 2.3.2 Fish

Ethical clearance was obtained from the ethical committee of Rufus Giwas Polytechnic, Owo, Nigeria before going for the fish sampling in February 2019 during the dry season. Two major types of fish species were selected because they are common (predominant) to River Owan, they are *Clarias anguillaris* popularly known as catfish, and *Oreochromis niloticus* known as tilapia fish which were obtained using nets with the help of fishermen in the community. Three fish of equal size and weight were collected for the two fish species, placed in an ice chest, and frozen at −20°C before dissection. Equal weight of their muscle tissues only was composited for the two fish species - *Clarias anguillaris* and *Oreochromis niloticus* which was used for this study. The fish samples were defrosted, cleaned, and washed thoroughly with distilled water before dissecting them through the tail to the spinal cord, and the muscle tissues were removed for each of the fish. The fish samples were assigned sample codes: F1 for *Clarias anguillaris* and F2 for *Oreochromis niloticus*. The samples were transferred to the lab in a labeled glass jar, kept at 4°C in a chest cooler, and then stored there. The fish samples were established by an aqua-culturist in the Institutions’ Fishery and Aquaculture Department (Rufus Giwa Polytechnic in Owo, Nigeria). *Clarias anguillaris* is known to be a bottom feeder and depends greatly on detritus while *Oreochromis niloticus* is a surface dweller (Adewumi et al., 2014; Wagaw et al., 2021).

### 2.4 Sediment sample preparation and treatment

A standard reference procedure for the analysis of PAH in sediment was employed (US EPA 8240 method). Samples of the sediment were air-dried in a dust-free laboratory at room temperature, sieved using a 2 mm mesh laboratory sieve, and packed into zip-lock bags. Ten grams (10 g) of the sieved sediment sample was carefully weighed into a vial meant for extraction that had first been washed using chromic acid and dried. A glass rod was used to properly mix 10 g of anhydrous sodium sulphate (Na_2_SO_4_) which was carefully weighed into the mixture. The sample was mixed with 50 mL (mL) of a 3:1 hexane and dichloromethane mixture (90 mL of n-hexane and 30 mL of dichloromethane made in a standard flask). The material was filtered after being shaken at 500 osc/min for 30 min on a shaker (Chen et al., 2007). The sample was then allowed to concentrate in the extraction bottle at room temperature for at least 24 h until about 2.0 mL of concentrated sample remained. Following this, n-hexane and dichloromethane were fractionated in an activated alumina (neutral)/silical gel column for effective clean-up. This extraction process was repeated twice. Using a rotary evaporator, the fraction was reduced to 1.0 mL volume. The aromatic extract was kept until analysis in dried organic free and chromic acid pre-cleaned glass vials with Teflon rubber closures in a freezer at −4.0°C.

### 2.5 Fish sample preparation and extraction

The United States Environmental Protection Agency standard for analytical procedures of 1986 was followed (USEPA, 1986). Using a whirling blender, the tissue from the fish sample was pulverized. One hundred (100) mL of acetone and n-hexane was added to a homogenizer cup along with five (5 g) of the fish sample. Samples were combined with 5 g of anhydrous sodium sulphate after being homogenized for 20 min at 100 rpm. A mixture of dichloromethane and n-hexane was used for the extraction using a Soxhlet extraction apparatus for about 5 h and evaporated to dryness the extracted solvent. The resultant extract was reconstituted using 50 mL n-hexane and later evaporated until 1.0 mL.

### 2.6 Sample analysis

A flame ionization detector (FID) in an Agilent 7890A GC was used for the instrumental analysis. One µL (1.0 µL) of the concentrated sample was injected into the column through a rubber septum using an exmire micro syringe in the splitless mode. Vapour separation occurs as a result of partitions between the liquid and gas phases. The following are the GC settings for analysis:

### 2.7 Gas chromatography operating conditions

The initial setting for the temperature of the GC oven was 45°C with a 2-min holding time. The temperature was raised to 240°C, while the gradient rate was 15°C/min, and the injector temperature was 280°C. After injecting samples, a gradient increase rate of 10 C/min allowed for the higher maximum working temperature to be reached, which was 300°C. The detector’s temperature was 340°C and the carrier gas used was helium. Pressure program (set point) at 14.0psi with an injection volume of 1.00 µL.

### 2.8 Gas chromatograph calibration

PAH standard comprising the 16 prioritized, 1-methylnaphthalene, and 2-methylnaphthalene PAHs of AccuStandard Inc. was purchased from a reputable commercial vendor in Nigeria and was used for the calibration. The GC temperature was set and allowed to increase gradually to reach optimum. Standards for calibration were set up in the range of 0.10 and 0.50 ppm (five levels) which were utilized for calibration. A standard of 1.00 µL volume was injected and run. The analytes’ coefficient of determination (*R* ^2^) for the calibration was all in the range of 0.995–0.999. Software for PAH calibration and quantification was employed, having a CLARITY-GC interface.

### 2.9 Source apportionment using diagnostic ratio

Diagnostic ratio (DR) is one of the techniques available to determine the origin of PAHs in environmental matrices qualitatively using the isomer ratios of the PAH components (Davis et al., 2019). It is applied as a result of the fact that PAH isomers possess analogous chemical characteristic behavior in the natural environment which is quite similar in terms of transformation and degradation. According to Tobiszewski *et al.* (2012) and Santos *et al.* (2017), the isomer ratios remain the same from the time of emission to the time of measurement. In order to recognize pyrogenic sources from petrogenic sources, people typically look at the ratios of Ant/(Ant + Phe) and Fla/(Fla + Pyr). A petroleum source is indicated by a ratio of Ant/(Ant + Phe) 0.1, but the dominance of combustion is indicated by a ratio of Ant/(Ant + Phe) > 0.1. Additionally, Fla/(Fla + Pyr) giving a value greater than 0.5 indicates combustion from biomass or coal sources, while ratio values between 0.4 and 0.5 indicate combustion from petroleum sources. Fla/(Fla + Pyr) of 0.4 ratio value is pointing to petroleum source. Tobiszewski *et al.* (2012) stated that a ratio of the LMW to HMW PAHs <1 shows a pyrogenic source, while >1 indicates a petrogenic source. According to Wang *et al.* (2010); Wang *et al.* (2017); and Chen *et al.* (2012), the ratio of BaA/(BaA + Chr) below 0.2 implies a petroleum source, but values in the range of 0.2–0.35 suggested petroleum source, especially liquid fossil fuel, vehicle, fuels, and crude oil, while values above 0.35 indicate the combustion of coal, grass, and wood.

### 2.10 Toxic equivalent factor (TEF), toxic equivalent quantity (TEQ), and lifetime risk assessment

The carcinogenic potential of individual PAHs (Cn) in comparison to that of Benzo[a]pyrene (BaP) is known as the toxic equivalent factor (TEF). As defined by the term “toxic equivalent quantity” (Nisbet and LaGoy, 1992):

$$\text{TEQ}=\sum C_{n}x{\text{TEF}}_{n}$$

In evaluating the lifetime risk assessment, several factors are responsible for the impact of pollutants such as PAHs on human health which include lifestyle, health condition, age, and contact time with the pollutant(s). Incremental lifetime cancer risks (ILCRs) were used to assess the lifetime risk of PAHs. Using Eqs. 1–4, the effects of three main exposure pathways - dermal, ingested, and inhaled were measured. In this study, two age groups - children (0–18 years) and adults (19–70 years) were taken into account. Accordingly, an ILCR of ≤10^–6^ indicates negligible risk, an ILCR of 10^–6^ to 10^–4^ indicates low risk, an ILCR of 10^–4^ to ≤10^–3^ indicates moderate risk, an ILCR of 10^–3^ to ≤10^–1^ indicates high risk, and an ILCR of ≥10^–1^ indicates extremely high risk (Yahaya et al., 2017; Qu et al., 2019; Xing et al., 2020).

BaPeq=∑Ci x TEF (1)

$$ILCRs\left(Dermal\right)=\frac{\text{CS}x\left(\text{CSF}\left(\text{Dermal}\right)\;\sqrt[{3}]{\left(\frac{\text{BW}}{70}\right)}\right)x\text{AF x SA}x\text{EF}X\text{ABS}x\text{ED}}{BW\;x\;AT\;x\;10^{6}}$$ (2)

$$ILCRs\left(Ingestion\right)=\frac{\text{CS}x\left(\text{CSF}\left(Ingestion\right)\;\sqrt[{3}]{\left(\frac{\text{BW}}{70}\right)}\right)x\text{IR}\left(\text{Ingestion}\right)x\text{EF}x\text{ED}}{BW\;x\;AT\;x\;10^{6}}$$ (3)

$$ILCRs\left(Inhalation\right)=\frac{\text{CS}x\left(\text{CSF}\left(\text{Inhalation}\right)\;\sqrt[{3}]{\left(\frac{\text{BW}}{70}\right)}\right)x\text{IR}\left(\text{Inhalation}\right)x\text{EF}x\text{ED}}{BW\;x\;AT\;x\;PEF}$$ (4)

The sum of converted PAHs for 7 CarPAHs based on toxic equivalents of BaP using the Toxic equivalent factor (TEF) by Nisbet and LaGoy (1992), the carcinogenic slope factor - CSF (mg/kg/day)^−1^, the body weight (BW) of the exposed resident (kg), the average lifespan (AT) in years, the exposure frequency (EF) in days per year, the exposure duration (ED) in years, others include the dermal adherence factor (AF) for sediment (mg/cm^2^/h), the dermal adsorption factor (ABS), the surface area of dermal exposure (SA) (cm^2^), the sediment ingestion rate (IR Ingestion) (mg/day), the inhalation rate (IR Inhalation) (m^3^/day), and the particle emission factor (PEF) (m^3^/kg). According to Peng *et al.* (2011), the CSF ingestion was taken as 7.30 (mg kg^−1^ day^−1^)^−1^, CSF for dermal as 25 (mg kg^−1^ day^−1^)^−1^, and CSF for inhalation to be 3.85 (mg kg^−1^ day^−1^)^−1^ of BaP based on the cancer-causing ability.

### 2.11 Statistical analysis and quality assurance

Excel and Minitab packages were employed for the descriptive data analysis, and generating the distribution charts. The GC was air-flushed before starting up to clean the column and get ready for fresh analysis. Quality control (QC) and blank samples were included within each batch of samples that were to be analyzed. Target compounds were not detected in the blank samples. High purity and analytical grade chemicals and reagents were used of >98.8% purity (including sodium chloride and anhydrous sodium sulphate). All solvents used (acetone (99.8%), dichloromethane (99.5%), n-hexane (99.8%), and acetonitrile) were GC grade and of high purity. The correlation coefficient (*r* ^2^) from the calibration curves was 0.995 indicating perfect linearity. Recovery studies of PAH surrogates ranged from 81.2% to 95.6%, and the spiked samples were between 79.7% and 91.5%. The signal-to-noise ratio of the examined blanks was multiplied by 3 and 10 to determine the limits of detection and quantitation (LOD and LOQ).

## 3 Results and discussion

### 3.1 PAHs concentration and distribution in the sediment samples

**FIGURE 2:**
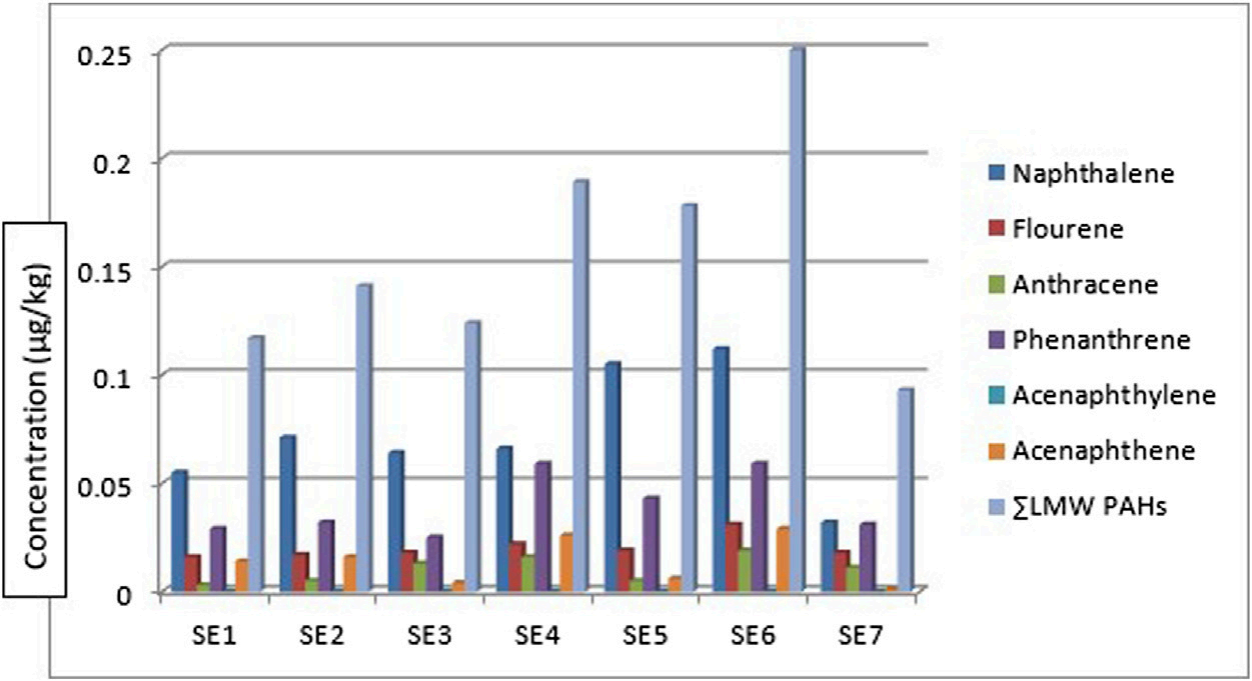
Concentration, comparison, and $\sum \text{LMW}{\text{PAH}}_{s}$ in the sediment samples.

Table 1 shows the concentrations of the various components of PAHs: the low molecular weight (LMW), high molecular weight (HMW), 1-methyl naphthalene, and 1-methyl naphthalene. All the LMW PAHs were detected in all the sediment samples except for acenaphthylene. The concentrations of naphthalene, fluorene, anthracene, phenanthrene, and acenaphthene were in the range: of 0.032—0.112 μg/kg, 0.031—0.016 μg/kg, 0.003—0.019 μg/kg, 0.025—0.059 μg/kg and 0.001—0.029 μg/kg respectively while their mean values were 0.072 μg/kg, 0.020 μg/kg, 0.010 μg/kg, 0.040 μg/kg and 0.014 μg/kg. Figure 2 gives the distribution of the low molecular weight PAHs across the seven sampling locations. The ∑LMW PAHs were in the range of 0.093—0.250 μg/kg. The highest concentration was from sediment sampling location 6 (SE6) (0.250 μg/kg) while the least was from sampling location 7 (SE7) (0.093 μg/kg). A publication by dos-Santos *et al.* (2018) reported total PAHs in sediment samples from the Estuary of Amazon River of Amapa of Brazil to be in the range of 22.20—158.90 ng/g. The values of naphthalene were in the range of 26.40–6.70 ng/g, acenaphthene 0.60–0.20 ng/g, anthracene was 1.00-ND, pyrene, and benzo[a]pyrene was 6.30-ND, and 8.80–0.60 ng/g. Almost all the sixteen PAHs analysed were detected from the study area. The total PAHs PAHes obtained were far greater than those of this study. Research conducted by Olayinka *et al.* (2019) on polycyclic aromatic hydrocarbons in sediment and biota (fish, crab, and shrimp) around Atlas Cove Nigeria revealed the presence of naphthalene with a mean concentration of 0.66 ± 0.01—1.17 ± 0.01 mg/kg, acenaphthylene: BDL—1.42 mg/kg while fluorene was only detected in one out of the 5 sampling locations with a concentration of 1.05 ± 0.053 mg/kg. The concentration of LMW PAHs in the sediment samples from Olayinka *et al.* (2019) was higher than the concentration obtained from this study. Gosai *et al.* (2018) reported mean ∑LMW PAHs higher than this study (1,437.499–65.36 and 3,024—179.36 ng/g) of sediment from two sampling locations along the Gujarat coastline.

**TABLE 1:** Concentration of ∑PAHs in various sediment and fish samples from rivers across the globe.

River	City/Country	∑PAHs in sediment (µg/kg dw)	∑PAHs in fish (µg/kg dw)	References
Around Atlas Cove	Nigeria	2.15–36.46	11.89–71.06	Olayinka *et al.* (2019)
Warri River	Nigeria	4587.7 (mean)	1098.5 (mean)	Asagbra *et al.* (2015)
Jhelum Riverine system	Pakistan	14.54–437.43	-	Raiz *et al.* (2019)
River Benue	Nigeria	55 ± 3–382 ± 9	-	Arowojolu *et al.* (2021)
Mediterranean sea	Egypt	13.5—22,600	-	Barakat *et al.* (2011)
Gulf of Suez	Egypt	1667.02–2671.27	621–4207 wet weight	Younis *et al.* (2018)
Amazon River	Brazil	22.2–158.9	-	Dos Santos *et al.* (2018)
Brisbane River	Australia	148–3079	-	Duodu *et al.* (2017)
Naples harbor	Southern Italy	9–31774	-	Sprovieri *et al.* (2007)
Jakarta bay	Indonesia	257–1511	-	Koike *et al.* (2012)
Langkawi Island	Malaysia	868–1637	-	Nasher *et al.* (2013)
Wei River	China	60.50—10,240.1	-	Pang *et al.* (2021)
Weihe River	China	362–15,667	-	Chen *et al.* (2015)
White Nile	South Sudan	566 to 674	566 to 674 wet weight	Abayi *et al.* (2021)
Guan River	China	43–169	-	He *et al.* (2014)
Mediterranean sea	Italy	40–679	-	Montuori and Triassi (2012)
Hooghly River	India	48–1831	-	Khuman *et al.* (2018)
Paraguacu River	Brazil	443.7–636.1	-	Santos *et al.* (2017)
Estero de Urias Estuary	Mexico	27–418	-	Jaward *et al.* (2012)
Delaware River	USA	3749–22,324	-	Kim *et al.* (2018)
Edremit Bay (Aegea Sea)	Turkey	0.65–175	-	Darilmax *et al.* (2019)
River Owan	Nigeria	0.28–0.81	ND-0.19	This study

**FIGURE 3:**
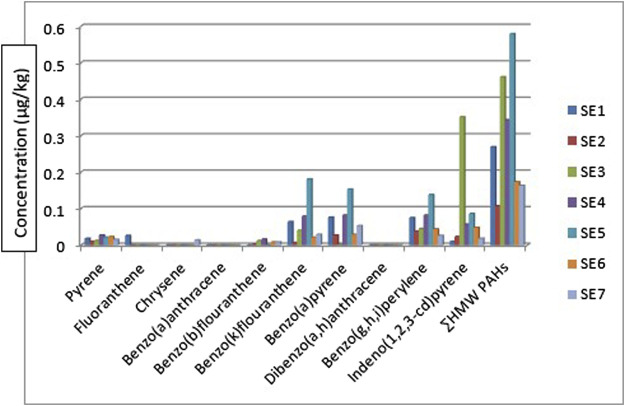
Concentration, comparison, and of $\sum \text{HMW}{\text{PAH}}_{s}$ in the sediment samples.

The HMW PAHs such as pyrene, benzo[b]fluoranthene, benzo[k]fluoranthene, benzo[a]pyrene, benzo[g,h, i]perylene, and indeno[1,2,3-cd]pyrene were detected in all the sediment sampling locations in the range: 0.010—0.027 μg/kg, BDL - 0.016 μg/kg, 0.007—0.181 μg/kg, 0.001—0.153 μg/kg, 0.026—0.138 μg/kg and 0.010—0.351 μg/kg respectively. Chrysene was only detected in sediment samples from sampling location 7 with a concentration of 0.014 μg/kg and fluoranthene was detected in sampling location 1 with a concentration of 0.026 μg/kg. Benz[a]anthracene and dibenzo[a,h]anthracene were below the detection limit (BDL) in all the sampling locations. The mean range concentrations of the sediment samples BDL - 0.085 μg/kg. Figure 3 gives the distribution of the high molecular weight PAHs in the sediment samples across the seven different sampling locations. The ∑HMW PAHs range was 0.107—0.579 μg/kg across the sampling locations. The highest concentration was from sampling location 5 (SE5) while the least was from sampling location 2 (SE2). High molecular weight PAHs determined by Olayinka *et al.* (2019) in sediments around Atlas Cove, Nigeria were below the detection limit of the equipment (0.001 mg/kg) with only one site having 4.97 ± 2.45, 3.03 ± 1.44, 2.08 ± 1.67, 4.10 ± 1.95 and 3.38 ± 1.89 mg/kg concentrations for chrysene, benzo[k]fluoranthene, benzo[b]fluoranthene, benzo[a]pyene and benzo[g,h, i]perylene respectively. In the same study (Olayinka et al., 2019), pyrene, fluoranthene, and benzo[a]anthracene are in the range of BDL—5.43 ± 0.18, 0.23 ± 0.02—4.73 ± 0.10 and BDL—2.48 ± 0.45 mg/kg.

**FIGURE 4:**
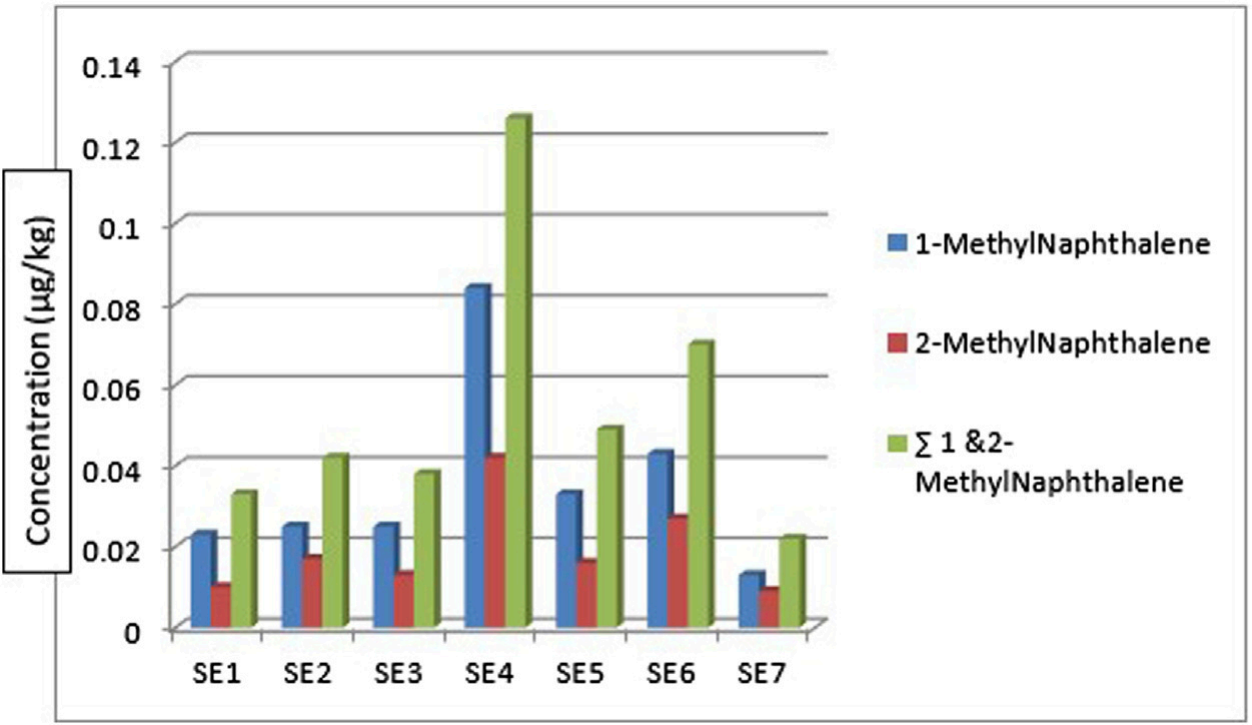
Concentration and ∑1 and 2-Methyl naphthalene ${\text{PAH}}_{s}$ in the sediment samples.

Figure 4 shows the concentration and distribution of 1-methyl naphthalene and 2-methyl naphthalene in the sediment samples across the seven different sampling locations. 1-methyl naphthalene and 2-methyl naphthalene were detected in the sediment samples from all the sampling stations. 1-methyl naphthalene is in the range: 0.013—0.084 μg/kg and 2-methyl naphthalene 0.009—0.042 μg/kg. The highest concentration of 1-methylnaphthalene and 2-methylnaphthalene was from the sediment sample from location 4 (SE4) at 0.126 μg/kg while the least was from sampling location 7 (SE7) at 0.022 μg/kg. The concentration of 1-methylnaphthalene and 2-methyl naphthalene obtained from sediment samples by Olayinka et al., 2019 around Atlas Cove, Nigeria was in the range of 0.19 ± 0.02—0.41 ± 0.02 mg/kg and 0.12 ± 0.00—0.27 ± 0.01 mg/kg for 1-methyl naphthalene and 2-methyl naphthalene respectively.

**FIGURE 5:**
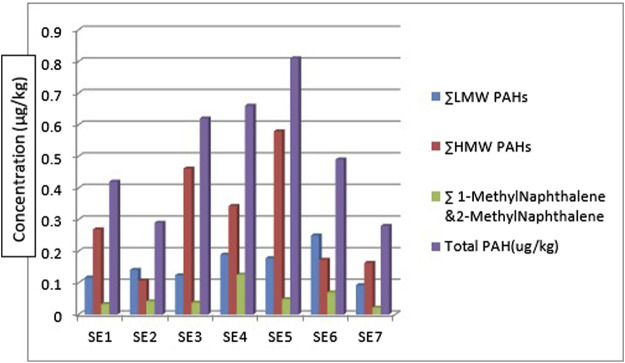
∑LMW , ∑HMW , and ∑1 and 2- Methyl naphthalene ${\text{PAH}}_{s}$ in the sediment samples.

Considering the ∑PAHs, the highest concentration is from the sediment sample from sampling location 5 (SE5) 0.810 μg/kg. The major contribution to this value is from the HMW components (∑HMW PAH) sampling location 5 (SE5) 0.579 μg/kg. The least ∑PAH components are from sediment samples from sampling location 7 (SE7) 0.280 μg/kg. The range of ∑PAHs in the sediment samples is 0.280–0.810 μg/kg (Concentration order is ∑PAHs SE5>SE4>SE3>SE6>SE1>SE2>SE7). Figure 5 shows the ∑LMW, ∑HMW, ∑1-methylnaphthalene, and 2-methylnaphthalene polycyclic aromatic hydrocarbons across all the sampling locations. These results are lower than what Olayinka *et al.* (2019) obtained from sediment samples around Atlas Cove, Nigeria which range between 2.15—36.46 mg/kg. Assessment of ∑16PAHs in the sediment of Wei River by Pang *et al.* (2021) was in the range of 60.50—10,241.10 ng/g while Raiz et al., 2019 reported 14.54—437.43 ng/g for PAHs sediment samples along Jhelum riverine system of lesser Himalayan region of Pakistan while a report published by Dos Santos *et al.* (2018) of Amazon River in Brazil reported a total PAHs concentration of 22.2—158.9 μg/kg of sediment samples from the river. Asagbra *et al.* (2015) determine the concentration of PAHs in sediment samples from the Warri River at Ubeji, Niger Delta, Nigeria with a mean concentration of 4587.70 ng/g. Barakat *et al.* (2011) also published the ∑PAH concentration of the Mediterranean Sea of Egypt to be in the range of 13.5—22,600 μg/kg. The total concentration of PAHs of sediment from the Delaware River in the USA river was reported by Kim *et al.* (2018) to be in the range of 3749–22,324 μg/kg. All these ∑PAH concentrations were higher than the results obtained from river Owan, Edo State, Nigeria (this study). Table 2 shows some other results obtained by other researchers across the globe. The presence of PAHs in the environment has been a contribution from a series of human activities and natural occurrences ranging from domestic to industrial sources. These include burning of fossil fuel, combustion of wood, vehicular emissions, incinerators, flaring gases from industries, deposition wildfires, leakages from pipelines, gasoline leakage from engine boats, etc. (Davis et al., 2019; D’Agostino et al., 2020). Some of the factors that could affect the concentrations of PAHs in the environment include the type of human activities in the study area (anthropogenic), natural occurrence (naturogenic), seasonal variations, sediment transport processes, physicochemical properties, etc. (D’Agostino et al., 2020; Ambade et al., 2023).

**TABLE 2:** PAHs in sediment samples.

PAH components/Sampling locations	SE1	SE2	SE3	SE4	SE5	SE6	SE7	SD	TEFs	ERL	ERM
**Naphthalene (NAT)**	0.055	0.071	0.064	0.066	0.105	0.112	0.032	0.003	0.001	160	2100
**Fluorene (FLR)**	0.016	0.017	0.018	0.022	0.019	0.031	0.018	0.001	0.001	19	500
**Anthracene (ANT)**	0.003	0.005	0.013	0.016	0.005	0.019	0.011	0.001	0.01	85	1100
**Phenanthrene (PHT)**	0.029	0.032	0.025	0.059	0.043	0.059	0.031	0.001	0.001	240	1500
**Acenaphthylene (ACL)**	BDL	BDL	BDL	BDL	BDL	BDL	BDL	-	0.001	44	640
**Acenaphthene (ACN)**	0.014	0.016	0.004	0.026	0.006	0.029	0.001	0.001	0.001	16	500
**∑LMW PAHs**	**0.117**	**0.141**	**0.124**	**0.189**	**0.178**	**0.25**	**0.093**				
**HMW PAHs**											
**Pyrene (PYR)**	0.018	0.010	0.012	0.027	0.021	0.023	0.015	0.001	0.001	600	5100
**Fluoranthene (FLT)**	0.026	BDL	BDL	BDL	BDL	BDL	BDL	0.001	0.001	665	2600
**Chrysene (CHR)**	BDL	BDL	BDL	BDL	BDL	BDL	0.014	-	0.01	380	2800
**Benz(a)anthracene (B[a]A)**	BDL	BDL	BDL	BDL	BDL	BDL	BDL	-	0.1	260	1600
**Benzo(b)fluoranthene (B[b]F)**	BDL	0.002	0.012	0.016	BDL	0.008	0.008	0.01	0.1	320	1880
**Benzo(k)fluoranthene (B[k]F)**	0.064	0.007	0.040	0.079	0.181	0.021	0.029	0.006	0.1	280	1620
**Benzo(a)pyrene (B[a]P)**	0.076	0.027	0.001	0.082	0.153	0.029	0.053	0.005	1	430	2800
**Dibenzo(a,h)anthracene (D[ah]A)**	BDL	BDL	BDL	BDL	BDL	BDL	BDL	-	1	63	260
**Benzo(g,h,i)perylene (B[ghi]P)**	0.075	0.038	0.045	0.082	0.138	0.044	0.026	0.004	0.01	85	330
**Indeno(1,2,3-cd)pyrene (IP)**	0.010	0.023	0.351	0.057	0.086	0.048	0.018	0.012	0.1	240	950
**∑HMW PAHs**	**0.269**	**0.107**	**0.461**	**0.343**	**0.579**	**0.173**	**0.163**				
**1-methylNaphthalene**	0.023	0.025	0.025	0.084	0.033	0.043	0.013	0.010	-	85	800
**2-methylNaphthalene**	0.010	0.017	0.013	0.042	0.016	0.027	0.009	0.010	-	70	670
**∑ 1-methylNaphthalene and2-methylNaphthalene**	**0.033**	**0.042**	**0.038**	**0.126**	**0.049**	**0.07**	**0.022**			**3442**	**24290**
**Total ∑PAH (ug/kg)**	**0.42**	**0.29**	**0.62**	**0.66**	**0.81**	**0.49**	**0.28**				
**∑TEQ**	**0.084608**	**0.031226**	**0.042003**	**0.09838**	**0.181,324**	**0.037584**	**0.061307**				

HMW, High molecular weight; LMW, Low molecular weight, SE1 to SE7—Sampling locations, SD, Standard deviation; TEF, Toxic equivalent factor; ERL, Effect range low; ERM, Effect range mean (Source: Nisbet and LaGoy, 1992; Adeniji et al., 2019).

### 3.2 PAHs concentration and distribution in fish samples

**FIGURE 6:**
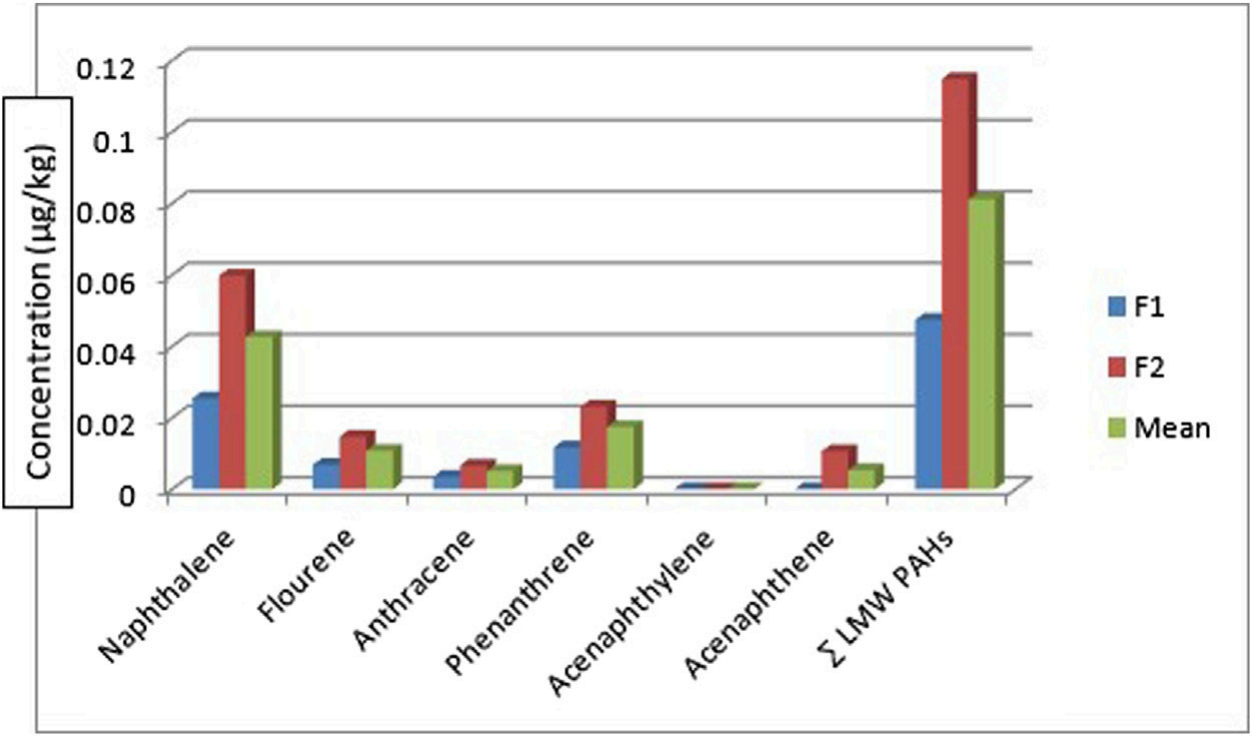
Concertration, mean, and $\sum \text{LMW }{\text{PAH}}_{s}$ in the fish samples.

Table 3 shows the concentration of PAHs in the fish samples. Catfish (*Clarias anguillaris)* (F1) and tilapia fish (*Oreochromis niloticus)* (F2) were the two types of fish analysed with varying concentrations of PAH components. Among the LMW PAHs, acenaphthylene was below the detection limit in the two fish samples. Acenaphthene was BDL in *Clarias anguillaris* (F1) but with a concentration of 0.0106 μg/kg in *Oreochromis niloticus* (F2). The concentrations of naphthalene, fluorene, anthracene, and phenanthrene for *Clarias anguillaris* (F1) were in the range of 0.0035–0.0254 μg/kg while for *Oreochromis niloticus* (F2) 0.0066–0.0598 μg/kg. The concentration of LMW PAHs is higher in (F2) 0.1148 μg/kg while F1 is 0.0474 μg/kg. Figure 6 is the chart showing the distribution of the LMW polycyclic aromatic hydrocarbons in the fish species. A study carried out by Usese *et al.* (2021) on the assessment of PAHs in Nile tilapia from Agboki Creek, Southwestern Nigeria obtained a concentration of 60.51—69.85 μg/kg which is higher than that of this study.

**TABLE 3:** PAHs in fish samples.

PAHs components	*Clarias anguillaris* (F1)	*Oreochromis niloticus* (F2)
LMW PAHs	(Ug/kg)	(Ug/kg)
**Naphthalene**	0.0254	0.0598
**Fluorene**	0.0068	0.0147
**Anthracene**	0.0035	0.0066
**Phenanthrene**	0.0117	0.0231
**Acenaphthylene**	BDL	BDL
**Acenaphthene**	BDL	0.0106
**∑ LMW PAHs**	**0.0474**	**0.1148**
**HMW PAHs**		
**Pyrene**	0.0057	0.0120
**Chrysene**	BDL	BDL
**Fluoranthene**	BDL	BDL
**Benzo(a)anthracene**	BDL	BDL
**Benzo(b)fluoranthene**	0.0013	0.0031
**Benzo(a)pyrene**	0.0058	0.0139
**Dibenzo(a,h)anthracene**	BDL	BDL
**Benzo(g,h,i)perylene**	BDL	0.0010
**Indeno(1,2,3-cd)pyrene**	0.0062	BDL
**Benzo(k)fluoranthene**	0.0033	0.0093
**∑ HMW PAHs**	**0.0225**	**0.0393**
**1-methylnaphthalene**	0.0112	0.0247
**2-methylnaphthalene**	0.0038	0.0106
**∑1and2-methylnaphthalene**	**0.015**	**0.0353**
**Total ∑PAHs (ug/kg)**	**0.08**	**0.19**
**∑LMW/∑HMW PAHs**	**2.107**	**3.921**
**EC recommendation**	**12.0 ug/kg**	

EC, european commission, Source, EC (2015).

**FIGURE 7:**
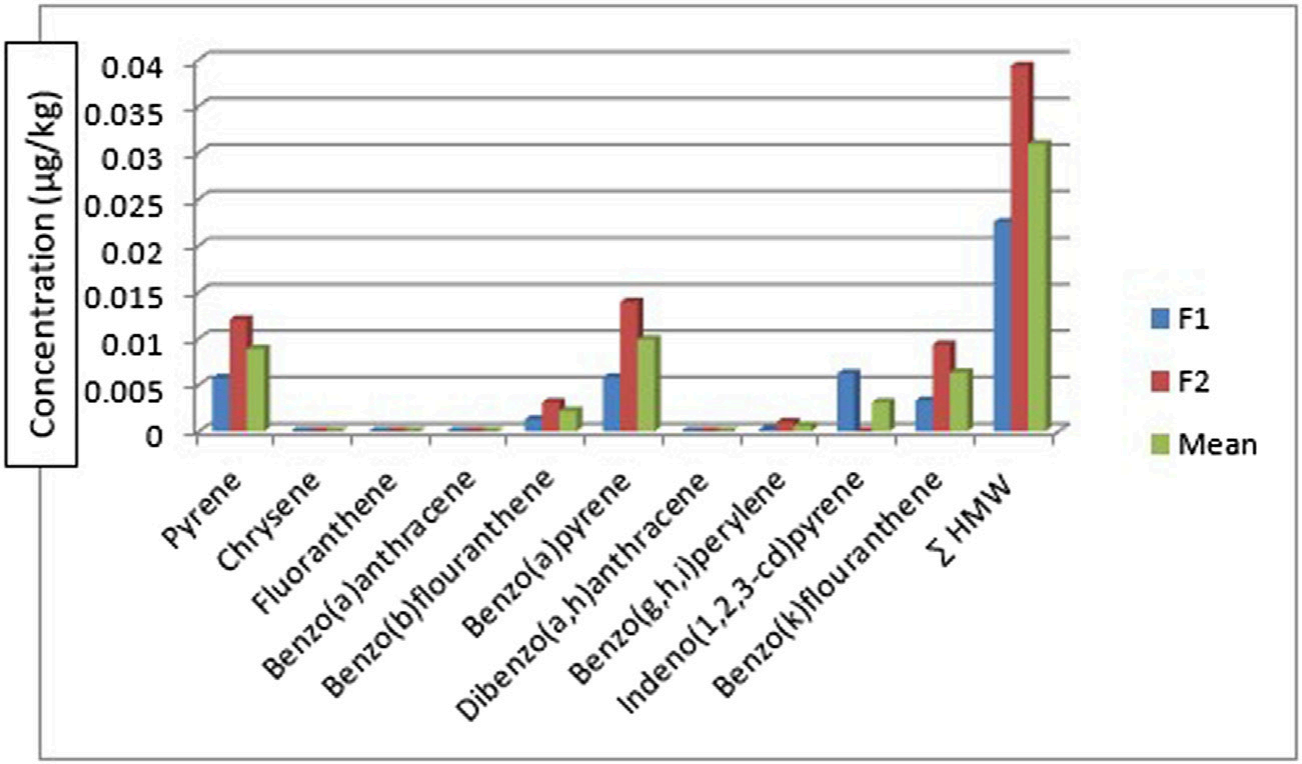
Concerntration, mean, and $\sum \text{HMW }{\text{PAH}}_{s}$ in the fish samples.

Figure 7 shows the distribution of the HMW polycyclic aromatic hydrocarbons in the fish species. The HMW PAHs such as chrysene, fluoranthene, benzo[a]anthracene, and dibenzo[a,h]anthracene were below the detection limit in the fish samples. The concentration of pyrene, benzo[b]fluoranthene, benzo[a]pyrene, benzo[g,h,i]perylene, and benzo[k]fluoranthene are of these concentrations for *Clarias anguillaris* (F1): 0.0057 μg/kg, 0.0013 μg/kg, 0.0058 μg/kg, BDL µg/kg, and 0.0033 μg/kg while that of *Oreochromis niloticus* (F2): 0.0120 μg/kg, 0.0031 μg/kg, 0.0139 μg/kg, 0.0010 μg/kg and 0.0093 μg/kg respectively. Indeno[1,2,3-cd]pyrene was BDL in *Oreochromis niloticus* but detected in *Clarias anguillarias* (F1) with a concentration of 0.0062 μg/kg. *Oreochromis niloticus* (F2) had the higher concentration of the HMW PAHs with a value of 0.0393 μg/kg while that of *Clarias anguillaris* (F1) was 0.0225 μg/kg. Benzo[a]pyrene was detected by Usese *et al.* (2021) in Nile Tilapia from Agboyi Creek, Southwestern Nigeria with a concentration of 0.08 ± 0.17 μg/kg. This value is higher when compared to this study. The high value recorded might be due to the level of pollution of the river which is in one of the industrialized cities of Nigeria (Lagos).

**FIGURE 8:**
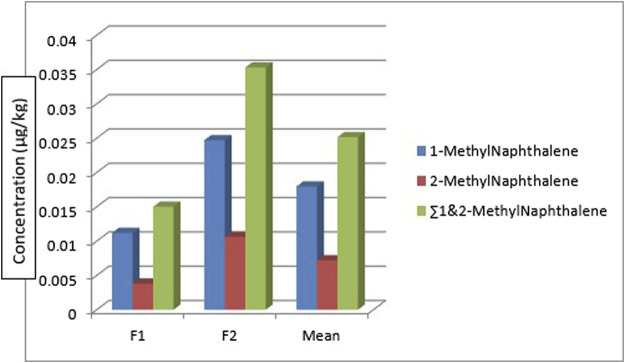
Concerntration, mean, and ∑1 and 2 Methyl naphthalene in the fish samples.

**FIGURE 9:**
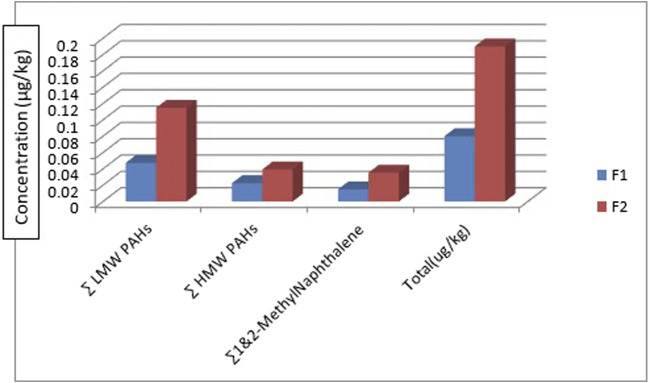
Summation and total concerntration of all the ${\text{PAH}}_{s}$ in the fish samples.

Figure 8 shows the distribution of the 1-methylnaphthalene and 2-methyl naphthalene in the two fish species. 1-methyl naphthalene and 2-methylnaphthalene are detected in the two fish species with a concentration of 0.0112 μg/kg and 0.0247 μg/kg of 1-methylnaphthalene in *Clarias anguillaris* (F1) and *Oreochromis niloticus* (F2) while that of 2-methylnaphthalene are 0.0038 μg/kg and 0.0106 μg/kg for the two fish species (F1 and F2). The ∑1-methylnaphthalene and 2-methylnaphthalene of *Oreochromis niloticus* are higher (0.0353 μg/kg) than *Clarias anguillaris* (0.0150 μg/kg). The ∑PAHs (LMW, HMW, 1-methylnaphthalene, and 2-methylnaphthalene as shown in Figure 9 are higher in *Oreochromis niloticus* (F2): 0.190 μg/kg than *Clarias anguillaris* (F1): 0.080 μg/kg (∑PAHs F2>F1). Figure 9 shows the distribution of ∑LMW, ∑HMW and ∑1-methylnaphthalene, and 2-methylnaphthalene in the fish samples. Asagbra *et al.* (2015) determined the concentration of PAHs in fish tissue from the Warri River at Ubeji, Niger Delta of Nigeria with a mean concentration of 1098.50 ng/g. The total concentration of 16 PAHs in fish samples reported by Younis *et al.* (2018) ranged from 621 to 4207 ng/g wet weight, with the species *Saurida undosquamis* having the highest concentration and *Stephanolepis diaspros* having the lowest from the Gulf of Suez, Egypt. Ekere *et al.* (2019) reported total ∑PAHs concentration of 2.626 and 2.061 μg/kg for catfish and tilapia fish from Rivers Niger and Benue confluence Lokoja, Nigeria. The ratio of LMW/HMW PAHs from this study is lower than what was reported by Ekere *et al.* (2019) of fish from Rivers Niger and Benue confluence, Lokoja, Nigeria. The concentration of PAHs in the fish samples analysed is lower than 12.0 μg/kg recommended regulatory limit by European Commission (2015).

### 3.3 Diagnostic ratio of PAHs in the sediment and possible sources

The result of the diagnostic ratio of the sediment samples from River Owan, Edo State is shown in Table 4 with their sources (petrogenic or pyrogenic). The percentage of petrogenic to pyrogenic in the ratio of Ant/(Ant + Phe) was 43% in the sediment. The ratio of Flt/(Flt + Pyr) in sediment was >0.1 meaning they are all from pyrogenic sources, showing 100%. The percentages of LMW/HMW PAHs of petrogenic to pyrogenic sources are in the range of 29%–71% in the sediment samples. Generally, petrogenic PAHs are characterized by the predominance of the two to three ring (LMW) PAHs, while pyrogenic PAHs are characterized by a high proportion of above 4-ring (HMW) PAHs (Wang et al.,2013). Furthermore, the ratio of the LMW/HMW <1 except perylene suggests pollution of pyrolytic origin according to Yan *et al.* (2009); and Tavakoly Sany *et al.* (2014). Microbial degradation also accounted for the resistance of HMW PAHs leading to a low LMW/HMW ratio according to Tavakoly *et al.* (2014). Chandru *et al.* (2008) also observed that a low ratio of the LMW/HMW could be caused by high volatility and solubility of LMW. The ratio of Flt/(Flt + Pyr) can also be used as an indicator of PAH origin. Flt/(Flt + Pyr) ratio <0.4 is attributed to a petrogenic source, while a ratio >0.5 suggests a pyrogenic source arising from wood and coal combustion (Yan et al.,2009; Hiller et al.,2011; Tavakoly Sany et al.,2014; Abayi et al.,2021). In this study, the ratio of Flt/(Flt + Pyr) > 0.5, indicates a pyrogenic source. A study by Zhao *et al.* (2017) used diagnostic ratio for PAHs sources in the sediment samples from a river and lake (Qinhuai River and Xuanwu Lake), results obtained revealed that sources were primarily from the burning of biomass and coal during the spring, fall, and winter. Other sites that were sampled revealed mixed sources of pyrogenic and petroleum sources such as this study. Furthermore, Gosai *et al.* (2018) reported PAHs sources in sediment samples from the Gujarat coastline using various isomeric ratios which revealed a mixed origin of petrogenic and pyrogenic sources.

**TABLE 4:** Diagnostic PAHS in sediment.

Locations	Ant/(Ant + Phe)	Flt/(Flt + Pyr)	LMW/HMW
**SE1**	0.1 Petrogenic	0.6 Pyrogenic	0.4 Pyrogenic
**SE2**	0.1 Petrogenic	0.8 Pyrogenic	1.3 Petrogenic
**SE3**	0.3 Pyrogenic	0.7 Pyrogenic	0.3 Pyrogenic
**SE4**	0.2 Pyrogenic	0.7 Pyrogenic	0.6 Pyrogenic
**SE5**	0.1 Petrogenic	0.7 Pyrogenic	0.3 Pyrogenic
**SE6**	0.2 Pyrogenic	0.7 Pyrogenic	1.5 Petrogenic
**SE7**	0.3 Pyrogenic	0.7 Pyrogenic	0.6 Pyrogenic

Ant–Anthracene, Phe—Phenanthrene, Flt–Fluoranthrene, Pyr–Pyrene, LMW, Low molecular weight, and HMW, High molecular weight.

### 3.4 Sediment quality guidelines (SQG)

Sediment quality guidelines (SQG) are one of the benchmarks employed to evaluate the level of pollution in an aquatic environment (Long and MacDonald, 1998; Darilmax et al., 2019). Comparing the concentrations of the PAHs in the sediment samples with SQG values, the total concentrations (0.280–0.810 μg/kg) were very low when compared with the regulatory values of effect range low and effect range mean (ERL and ERM) for all the sampling stations (Table 1). This shows that no toxic effect occurrence is possible in this area at present. Also, looking at the concentrations of PAHs around the globe from rivers of other sediments across the globe (Table 2), the concentrations from this study are low. A study by Darilmax *et al.* (2019) reported a concentration range of 0.65—175 ng/g of surficial sediments of Edremit Bay (Aegean Sea). The least or minimum value (0.65 ng/g) from Darilmax’s team is in the range of concentrations recorded in this study (0.28—0.81 μg/kg). Ambade *et al.*, 2022a also reported values that were low with fewer ecological hazards of sediment samples from the Estuary of the Subarnarekha India. It is important to note that the presence of PAHs in the ecosystem has negative biological implications on various biological species and causes a change in the proper functioning of these organisms from long-term exposure (Akhbarizadeh et al., 2016). Some of the potential human health implications of the presence of PAHs include the capability of causing cancer, mutagenicity, teratogenic effects, damage to the central nervous system (CNS), and so on. Hence, constant monitoring is needed to assess the environmental risk to wildlife and human health. Proper environmental management including a waste disposal plan using the best available practices (bap) should be employed. In addition, indiscriminate disposal of sewage and refuse into the river should be outlawed (Davis et al., 2019; D’Agostino et al., 2020; Ambade et al., 2022b; Ambade et al., 2023).

### 3.5 ILCR assessment

Looking at the result from the ILCRs evaluated, children and adults are the age groups considered with three routes of exposure (dermal, ingestion, and inhalation). The results of the ILCR are presented in Table 5. The results are in the following range for children’s dermal, ingestion, and inhalation 9.53 × 10^−8^ to 1.46 × 10^−6^, 2.16 × 10^-7^to 3.33 × 10^−6^, and 9.15 × 10^−12^ to 1.41 × 10^−10^ respectively for all the sampling sites. For the adult, the results were in the range of 2.04 × 10^−7^ to 3.13 × 10^−6^, 1.15 × 10^−7^ to 1.76 × 10^−6^, and 7.78 × 10^−12^ to 1.20 × 10^−10^ respectively for dermal, ingestion and inhalation. These range values for the three routes of exposure, and the two age groups, ILCR implications show negligible risks. A study reported by Bhutto *et al.* (2021) gave ILCRs for children: 9.51 × 10^−2^ -2.03 × 10^−5^ with an average of 4.76 × 10^−2^ and 4.01 × 10^−2^—8.57 × 10^−6^ with an average of 2.01 × 10^−2^ for adult indicating a moderate cancer risk to children and threat to adult. Incremental lifetime cancer risks of two locations (ASSBRY and NAV) published by Gosai *et al.* (2018) ranged from 4.11 × 10^−6^ - 2.11 × 10^−5^ to 9.08 × 10^−6^–4.50 × 10^−3^ implying cancer risk at a higher level at NAV when compared to ASSBRY.

**TABLE 5:** Incremental life cancer risk (ILCR) assessment.

Age groups/Routes	SE1	SE2	SE3	SE4	SE5	SE6	SE7	Mean	Risk implication
**ILCR (Children) Dermal**	7.28E-07	2.69E-07	9.53E-08	8.06E-07	1.46E-06	2.95E-07	4.94E-07	5.93E-07	Negligible
**ILCR (Adult) Dermal**	2.55E-07	5.74E-07	2.04E-07	1.72E-06	3.13E-06	6.30E-07	1.06E-06	1.08E-06	Negligible
**ILCR (Children) Ingestion**	1.65E-06	6.10E-07	2.16E-07	1.83E-06	3.33E-06	6.69E-07	1.12E-06	1.35E-06	Negligible
**ILCR (Adult) Ingestion**	8.76E-07	3.23E-07	1.15E-07	9.70E-07	1.76E-06	3.55E-07	5.94E-07	7.14E-07	Negligible
**ILCR (Children) Inhalation**	6.99E-11	2.58E-11	9.15E-12	7.74E-11	1.41E-10	2.83E-11	4.74E-11	5.69E-11	Negligible
**ILCR (Adult) Inhalation**	5.94E-11	2.19E-11	7.78E-12	6.58E-11	1.20E-10	2.41E-11	4.03E-11	4.84E-11	Negligible

## 4 Conclusion

This study showed that LMW PAHs are more predominant than HMW PAHs for the sediment samples from River Owan. Meanwhile, their concentrations vary from one sampling location to the other. The levels of ∑PAHs in the fish samples are in varying concentrations, LMW PAHs were higher than the HMW PAHs in the fish samples. The concentration of PAHs in *Clarias anguillaris* (catfish) is higher than that of *Oreochromis niloticus* (tilapia fish). The concentration of ∑PAHs in the fish samples analysed is far lesser than the European Commission regulation limit. The result of the diagnostic ratio of the sediment samples shows that the sources are more pyrogenic than petrogenic sources. Sediment quality assessment revealed that the total concentrations of PAHs are lower than the regulatory values of SQG (ERL and ERM) for all the sampling stations. This shows that no toxic effect occurrence in this area at present. ILCR assessment evaluated showed negligible risk. To prevent any potential ecotoxicological problems in the near future, constant monitoring of PAH levels in the study area will be required. In addition, assessment of PAHs during the raining season and increasing the frequency of sampling is of interest for further study.

## Data Availability

The original contributions presented in the study are included in the article/supplementary material, further inquiries can be directed to the corresponding author.
